# Relationship of workplace exercise with work engagement and psychological distress in employees: A cross-sectional study from the MYLS study

**DOI:** 10.1016/j.pmedr.2019.101030

**Published:** 2019-12-09

**Authors:** Takashi Jindo, Yuko Kai, Naruki Kitano, Kenji Tsunoda, Toshiya Nagamatsu, Takashi Arao

**Affiliations:** a Physical Fitness Research Institute, Meiji Yasuda Life Foundation of Health and Welfare, 150 Tobuki, Hachioji, Tokyo 192-0001, Japan; b Yamaguchi Prefectural University, 3-2-1 Sakurabatake, Yamaguchi, Yamaguchi 753-8502, Japan; c Yamano College of Aesthetics, 530 Yarimizu, Hachioji, Tokyo 192-0396, Japan

**Keywords:** MYLS, Meiji Yasuda Lifestyle, PA, physical activity, SB, sedentary behavior, OR, odds ratio, UWES-9, Utrecht work engagement scale, K6, Kessler Psychological Distress Scale, METs, Metabolic equivalents, BMI, body mass index, Physical activity, Sedentary behavior, Mental health, Work productivity, Worker

## Abstract

We investigated the relationship between workplace exercise and psychological distress, and work engagement, both of which are factors related to the mental health and work productivity of employees. Data from the Meiji Yasuda Lifestyle study, collected from July 2017 through December 2017, were used. Data from an annual health checkup and questionnaire were collected from the Meiji Yasuda Shinjuku Medical Center in Tokyo, Japan. The 1321 participants (mean age: 50.8 ± 9.5 years, rate of female: 68.2%, rate of white-collar workers: 64.7%) were divided into three groups based on the frequency of performing workplace exercise: less than once, once or twice, and three or more times a week. Vigor of work engagement and psychological distress were assessed using the Utrecht Work Engagement Scale and Kessler Psychological Distress Scale (K6). Logistic regression analyses were performed to examine relationships between workplace exercise frequencies and work engagement or psychological distress. Demographic variables, health behaviors, health status, work characteristics, and objectively measured physical activity and sedentary behavior were adjusted for odds ratio (OR) and 95% confidence interval (95% CI). Participants who performed workplace exercise once or twice and three or more times a week showed a significantly higher OR (OR = 1.93, 95% CI = 1.00–3.71, p = 0.049, OR = 1.63, 95% CI = 1.23–2.15, p = 0.001, respectively) for vigor of work engagement. Neither groups of workplace exercise showed a significant OR for psychological distress even when adjusted for covariates. The practice of workplace exercise is positively and independently related to vigor of work engagements of physical activity and sedentary behavior, and the association was observed regardless of the frequency of once or twice, or three or more times a week. Workplace exercise, however, does not correlate with psychological distress. Our findings indicated that workplace exercise at least once or twice a week could have practical implications for the enhancement of vigor of work engagement, especially among white-collar workers.

## Highlights

• Practice of workplace exercise was positively related to work engagement.
• Relationship of workplace exercise with work engagement was independent of physical activity and sedentary behavior.
• Workplace exercise, however, might not improve psychological distress.

## 1 Introduction

The prevention of mental disorders (e.g., depression) and promotion of employee mental health are important issues given their relationships with individual productivity at the workplace (Boles et al., 2004). As a result, numerous employers currently devote substantial resources to promote work engagement, given its positive impact on both productivity and employee health status. Work engagement is defined as a positive, fulfilling, work-related state of mind that is characterized by vigor, dedication, and absorption (Schaufeli et al., 2002). Previous studies report that high-work engagement is related to productivity (Salanova et al., 2005, Xanthopoulou et al., 2009) and mental and physical health (Eguchi et al., 2015, Schaufeli et al., 2008). Moreover, work engagement can be a predictor of the future incidence of depression (Imamura et al., 2016).

Regular daily exercise is positively associated with work engagement (Nishi et al., 2017). Thus far, there is very limited research elucidating the relationships between work engagement and workplace exercises, which are any exercises implemented at a workplace for the purpose of health promotion (Knight et al., 2017, Michishita et al., 2017). Workplace exercise may enhance not only employees’ health but also improve factors associated with work engagement such as social support from workplace colleagues or supervisors. A recent interventional study (Michishita et al., 2017) reported that workplace exercise programs, when held during lunch-time, improved the vigor of work engagement, whereas adherence to exercise programs decreased when employees engaged in atypical work activities, such as a business travel. Another interventional study (Strijk et al., 2013) investigated the effectiveness of an aerobic and yoga centered-program for hospital workers based on their compliance with the program. Vigor of work engagement improved only for participants who showed high compliance with the exercise program. Many individuals discontinue participation in such programs due to lack of time (Atlantis et al., 2004). The problems of low adherence or attrition may be due to practical limitations. Therefore, in non-interventional work settings, the planning of workplace exercise programs should investigate the relationships between the frequency of workplace exercise and work engagement from a practical point of view. Several recent studies have reported that physical activity (PA) and sedentary behavior (SB) are factors related to for work engagement (Ishii et al., 2018, Munir et al., 2015, Nishi et al., 2017), suggesting that improvement in PA or SB enhances work engagement. However, to the best of our knowledge, there is no information regarding the effect of workplace exercise on work engagement and psychological distress in non-interventional work settings that involve participants with various job types, work-place characteristics, health status, or health behaviors.

Thus, the purpose of this study was to investigate the specific relationship between workplace exercises, focusing on the frequency of practice, vigor of work engagement, and psychological distress among employees.

## 2 Methods

### 2.1 Study procedure and participants

We incorporated data from the Meiji Yasuda Lifestyle (MYLS) longitudinal cohort study established in 2013. The study was conducted at the Meiji Yasuda Shinjuku Medical Center in Tokyo, Japan, and included annual health checkup and questionnaire data. Participants provided informed consent to use their health checkup data for research. The Ethical Committee of the Meiji Yasuda Life Foundation of Health and Welfare approved this study protocol (approval no. 28006).

Participants included 1954 employees, and data were obtained from July 2017 through December 2017. Participants worked in multiple companies belonging to a single health insurance provider. Employees who did not allow the use of their data (n = 64), who did not agree to wear the accelerometer, did not complete the measurement (n = 534), who had a history of diagnosed mental illness including depression (n = 33), and who had no retrievable medical history (n = 2) were excluded from the study. According to the Kessler Psychological Distress Scale (K6) scale, 28 participants were judged to have severe psychological distress based on assessment precedent set by Nishi et al. (2018). Those participants, however, were included in the analysis because they did not have a history of mental illness. Data from the remaining 1321 participants were used for analysis.

### 2.2 Frequency of workplace exercise

The frequency of performing workplace exercise was assessed by asking participants “Do you perform exercise or calisthenics at the workplace during weekly working hours?” Any type of exercise (i.e., supervision by an expert or not, exercise type, or duration) was included in the workplace exercise category. Participants who performed workplace exercise also identified the frequency of exercise sessions per week. Participants were divided into three groups based on their answer: less than once a week, once or twice a week, or three or more times a week. The frequency categories were established based on workplace exercise interventions reported in previous studies that conducted sessions once per week (Strijk et al., 2013) or three to four times a week (Michishita et al., 2017).

### 2.3 Vigor of work engagement and psychological distress

Vigor of work engagement was assessed using the shortened Japanese version of the Utrecht Work Engagement Scale (UWES-9) (Schaufeli et al., 2006, Shimazu et al., 2008). The scale consists of three subscales with nine items that assess the current positive mental state for work such as vigor, dedication, and absorption. This scale has high internal reliability (α = 0.91–0.92) and good test–retest reliability (ICC = 0.66) (Shimazu et al., 2008). Here we used a vigor subscale of three items. Previous research confirms that each of these three subscales are strongly correlated with one another (r = 0.75–0.98) (Schaufeli et al., 2006). Therefore, the vigor subscale should reflect the overall work engagement. Because there is no validated cut-off score for this scale, data were dichotomized into low (≤8 points) and high (≥9 points) groups by the median of all participants. The cut-off score of 8 points is equivalent to the average score from a large scale data set of Japanese employees (n = 6377) (Shimazu et al., 2010). This report showed that the average score of Japanese employees was the lowest among the 16 countries included in the study, thereby the employees with ≤8 points were assumed to have an actual low work engagement.

Psychological distress was evaluated using the Japanese version of the K6 scale (Furukawa et al., 2008, Kessler et al., 2002). This scale is comprised of six psychological distress items such as nervousness or restless occurring within the previous month. The response option range is from 0 to 4 (none of the time, or all of the time), and the total score range is from 0 to 24. The scale has a high accuracy for diagnosing mood and anxiety disorders (AUC = 0.94) (Furukawa et al., 2008). For our analysis, data were dichotomized by 5 points of the score, which is used as the cut-off value for moderate psychological distress (Nishi et al., 2018).

### 2.4 Physical activity and sedentary behavior

PA and SB were evaluated using a triaxial accelerometer and an epoch length of 60-seconds (Active style Pro HJA-750C; Omron Healthcare Co. Ltd., Kyoto, Japan). The Active style Pro is highly accurate (r = 0.88), with total energy expenditure measured using a double-labeled water method in a free-living condition (Murakami et al., 2016). In addition, the device is an updated model of the Active style Pro HJA-350IT and has a comparable accuracy for detecting SB with the ActiGragh™ GT3X+ (ActiGraph LLC, Pensacola, Florida, USA) (Kurita et al., 2017) that is commonly used for SB research.

The accelerometer was mailed with activity measurement instructions and a health checkup kit 2 weeks prior to the health checkup day for each participant. Participants were instructed to continuously wear the accelerometer on their waist while they were awake, except while swimming or bathing, until the date of their health checkup. Non-wear time was defined as an interval of ≥20 consecutive minutes under the detectable intensity of the accelerometer (i.e., no signal). Every recorded intensity without non-wear time was aggregated, and that sum was treated as wearing time. Valid days were defined as days when the participants had ≥ 10 h of wearing time (Masse et al., 2005). Data from participants who had ≥ 4 valid days during the measurement period were included in the analysis (Trost et al., 2005). The time spent in SB was defined as the intensity of ≤1.5 metabolic equivalents (METs) according to the widely accepted definition (Bames et al., 2012). Because intensity and duration of workplace exercise were not evaluated, PA was defined as the intensity of ≥1.6 METs (light to vigorous activities) (Ainsworth et al., 2011) in a complete day. The variables of SB and PA were used as continuous variables (min/day) in the analyses.

### 2.5 Demographic variables

Demographic variables included age, sex, body mass index (BMI), educational level (≤ senior high school, ≥college/junior college), subjective economic status (very poor, poor, good, very good), marital status (married, unmarried), health status (BMI, subjective sleep quality; very bad, bad, good, very good), health behaviors (daily alcohol consumption; never, <23.0 g, ≥23.0 g, smoking status; not smoke, smoke), job type (administrative, office clerk, professional, service or sales, others), hiring status (regular employee, others), and average overtime hours (not applicable, 0–5 h, 5–10 h, ≥10 h/week). All demographic variables except BMI were assessed through a self-reported questionnaire. BMI was calculated using height and weight data from the health checkup.

### 2.6 Statistical analysis

Descriptive statistics of the mean ± standard deviation for continuous variables and number and percentage for ordinal and nominal variables were calculated. Because 27.6% of participants had missing data in the questionnaire (i.e., invalid or no answer) or accelerometer (i.e., too few valid days’ worth of data), these missing data were input in accordance with the hypothesis of missing at random (i.e., the missing value do not depend on the variable itself but on some other observed variables). Twenty multiple imputed data sets were prepared, and pooled results from separately integrated results were analyzed by applying Rubin’s rules (Raghunathan et al., 2001, Schafer and Graham, 2002). To examine the relationships between the frequency of workplace exercise and vigor of work engagement or psychological distress, a logistic regression analysis was adopted using work engagement and psychological distress as objective variables; the frequency of workplace exercise as the explanatory variable; and demographics, health status, work characteristics, and health behavior variables as the covariates. The odds ratio (OR) and 95% confidential interval (95% CI) for vigor of work engagement and psychological distress were calculated based on three multivariable-adjusted models (models 1–3). Model 1 was adjusted for age and sex; model 2 was incorporated into model 1 for BMI, educational level, subjective economic status, marital status, daily alcohol consumption, smoking status, subjective sleep quality, job type, hiring status, and average overtime hours; and in model-3, to consider the effect of PA and SB in daily life, the PA and SB were incorporated into model 2. The not-imputed data, which included the complete data with no missing values, were also analyzed using the same logistic regression models.

In addition, stratified analysis by job type was conducted using the same logistic regression models to confirm whether the job types modulate the association between workplace exercise and outcomes. To maintain sample size in the analysis, job type was re-categorized into office clerk, service or sales, and administrative plus professional. Additionally, because there was a limited number of participants who performed workplace exercise once or twice a week, they were combined with the group of three or more times a week.

The SPSS version 25.0 (IBM, Inc., Armonk, NY) was used for data analysis. The level of statistical significance was set at *P* < 0.05.

## 3 Results

Table 1 shows participants’ characteristics in the imputed and not-imputed data. One or more missing data were observed in 12 variables, except for age, sex, BMI, smoking, and alcohol consumption status. With these 12 variables, missing data were observed for 0.2–14.5% of participants. At least one missing data variable was found in 27.6% of participants. The number and percentage of participants in each frequency of the workplace exercise group was as follows: less than once (n = 515, 39.0%), once or twice (n = 56, 4.2%), and three or more times (n = 750, 56.8%) a week. The rate of white-collar workers, such as administrative, office clerk, and professional, comprised more than half of the participants (n = 854, 64.7%).

**Table 1:** Comparison of participants’ characteristics between imputed and not-imputed data.

Variables	Imputed data (n = 1321)	Complete data (n = 956)	Missing rate
Age, years old	50.8 ± 9.5	50.8 ± 9.2	0 (0.0)

*Sex*			
Male	420 (31.8)	308 (32.2)	0 (0.0)
Female	901 (68.2)	648 (67.8)	
Body mass index, kg/m^2^	23.1 ± 3.8	23.0 ± 3.7	0 (0.0)

*Educational level*			
≦Senior high school	378 (28.6)	268 (28.0)	56 (4.2)
≧College/junior college	943 (71.4)	688 (72.0)	

*Economic status*			
Very poor	57 (4.3)	43 (4.5)	36 (2.7)
Poor	327 (24.8)	233 (24.4)	
Good	853 (64.6)	623 (65.2)	
Very good	84 (6.3)	57 (6.0)	

*Marital status*			
Married	843 (63.8)	608 (63.6)	26 (2.0)
Unmarried	478 (36.2)	348 (36.4)	

*Daily alcohol consumption*			
Never	255(19.3)	174 (18.2)	0 (0.0)
<23.0 g	808(61.2)	599 (62.7)	
≧23.0 g	258(19.5)	183 (19.1)	

*Smoking status*			
Not smoke	1082 (81.9)	791 (82.7)	0 (0.0)
Smoke	239 (18.1)	165 (17.3)	

*Sleep quality*			
Very bad	92 (7.0)	60 (6.3)	3 (0.2)
Bad	545 (41.2)	396 (41.4)	
Good	583 (44.1)	419 (43.8)	
Very good	101 (7.7)	81 (8.5)	

*Job type*			
Administrative	219 (16.6)	165 (17.3)	60 (4.5)
Office clerk	520 (39.4)	400 (41.8)	
Professional	115 (8.7)	84 (8.8)	
Service or sales	436 (33.0)	290 (30.3)	
Others	30 (2.3)	17 (1.8)	

*Hiring status*			
Regular employee	1078 (81.6)	772 (80.8)	48 (3.6)
Others	243 (18.4)	184 (19.2)	

*Average overtime hours*			
Not applicable	147 (11.1)	101 (10.6)	123 (9.3)
0–5 h/week	602 (45.6)	444 (46.4)	
5–10 h/week	253 (19.1)	178 (18.6)	
≧10 h/week	319 (24.2)	233 (24.4)	
Physical activity, min/day	340.9 ± 91.6	338.3 ± 90.0	191 (14.5)
Sitting time, min/day	536.0 ± 109.3	540.3 ± 108.8	191 (14.5)

*Frequency of workplace exercise*			
Less than once/week	515 (39.0)	384 (40.2)	46 (3.5)
Once or twice/week	56 (4.2)	41 (4.3)	
More than 3 times/week	750 (56.8)	531 (55.5)	

*Work engagement*			
≦8 point	593 (44.9)	431 (45.1)	58 (4.4)
≧9 point	728 (55.1)	525 (54.9)	

*K6*			
≦4 point	948 (71.8)	687 (71.9)	8 (0.6)
≧5 point	373 (28.2)	269 (28.1)	

Values are numbers (percentages) except age, body mass index, physical activity, sedentary behavior.

Table 2 shows characteristics of participants in each frequency group of workplace exercise. Variables that displayed significant group differences among all multiple imputed datasets were age, sex, BMI, educational level, marital status, job type, hiring status, average overtime working hours, PA, and vigor of work engagement.

**Table 2:** Characteristics of participants in each group.

Variables	(1) Less than once/week (n = 515)	(2) Once or twice/week (n = 56)	(3) More than 3 times/week (n = 750)	Average *P*-value of multiple imputed datasets (range of the values)
Age	49.2 ± 9.1	49.3 ± 9.1	52.0 ± 9.6	<0.001 (<0.001)

*Sex*				
Male	159 (30.9)	35 (62.3)	226 (30.1)	<0.001 (<0.001)
Female	356 (69.1)	21 (37.7)	524 (69.9)	
Body mass index, kg/m^2^	22.6 ± 4.0	23.2 ± 2.9	23.4 ± 3.7	0.002 (<0.001–0.008)

*Educational level*				
≦Senior high school	133 (25.8)	4 (7.9)	241 (32.1)	<0.001 (<0.001)
≧College/junior college	382 (74.2)	52 (92.1)	509 (67.9)	

*Economic status*				
Very poor	21 (4.1)	2 (3.7)	34 (4.5)	0.270 (0.113–0.422)
Poor	146 (28.4)	13 (22.5)	168 (22.4)	
Good	321 (62.3)	38 (67.9)	495 (65.9)	
Very good	27 (5.2)	3 (5.9)	54 (7.2)	

*Marital status*				
Married	306 (59.4)	39 (70.1)	498 (66.3)	0.029 (0.009–0.053)
Unmarried	209 (40.6)	17 (29.9)	253 (33.7)	

*Daily alcohol consumption*				
Never	108 (21.0)	5 (9.7)	141 (18.8)	0.032 (0.011–0.074)
<23.0 g	324 (63.0)	35 (62.4)	449 (59.8)	
≧23.0 g	82 (15.9)	16 (27.9)	160 (21.4)	

*Smoking status*				
Not smoke	434 (84.2)	42 (74.2)	607 (80.9)	0.101 (0.038–0.188)
Smoke	81 (15.8)	14 (25.8)	143 (19.1)	

*Sleep quality*				
Very bad	39 (7.6)	2 (3.8)	51 (6.8)	0.168 (0.102–0.273)
Bad	232 (45.0)	26 (47.0)	287 (38.2)	
Good	211 (40.9)	25 (43.9)	348 (46.3)	
Very good	34 (6.5)	3 (5.4)	65 (8.6)	

*Job type*				
Administrative	82 (15.8)	14 (25.6)	123 (16.5)	<0.001 (<0.001)
Office clerk	273 (53.1)	16 (27.8)	232 (30.9)	
Professional	87 (16.8)	10 (18.7)	18 (2.3)	
Service or sales	59 (11.5)	14 (25.5)	363 (48.4)	
Others	14 (2.7)	1 (2.4)	15 (2.0)	

*Hiring status*				
Regular employee	391 (75.9)	47 (84.2)	640 (85.3)	<0.001 (<0.001–0.001)
Others	124 (24.1)	9 (15.8)	111 (14.7)	

*Average overtime working hours*				
Not applicable	27 (5.2)	5 (9.5)	115 (15.3)	<0.001 (<0.001)
0–5 h/week	269 (52.2)	22 (39.4)	312 (41.5)	
5–10 h/week	102 (19.8)	7 (12.1)	144 (19.2)	
≧10 h/week	118 (22.8)	22 (39.1)	180 (24.0)	
Physical activity, min/day	332.9 ± 92.5	307.7 ± 95.1	348.8 ± 89.7	0.001 (<0.001–0.005)
Sitting time, min/day	541.7 ± 105.2	559.2 ± 130.3	530.4 ± 110.0	0.066 (0.006–0.285)

*Work engagement*				
≦8 point	288 (55.9)	21 (37.5)	284 (37.9)	<0.001 (<0.001)
≧9 point	227 (44.1)	35 (62.5)	466 (62.1)	

*K6*				
≦4 point	350 (68.0)	41 (73.6)	557 (74.3)	<0.048 (0.017–0.086)
≧5 point	165 (32.0)	15 (26.4)	193 (25.7)	

Table 3 shows the results of the logistic regression analysis with the relationship between the frequency of workplace exercise and vigor of work engagement or K6 scores. The group of those who exercised three or more times a week displayed significantly higher OR (OR = 1.63, 95% CI = 1.23–2.16, p = 0.001) for vigor in every analytical model, whereas the group of those who exercised once or twice a week had a significant OR for vigor in model 1 (OR = 1.91, 95% CI = 1.04–3.49, p = 0.036) and model 3 (OR = 1.93, 95% CI = 1.00–3.71, p = 0.049).

**Table 3:** Comparison of work engagement and psychological distress between the groups.

Frequency of workplace exercise	Model 1^a^	Model 2^b^	Model 3^c^
	OR 95% CI	OR 95% CI	OR 95% CI
*Work engagement (odds ratio to high vigor)*			
Less than once/week	Ref	Ref	Ref
Once or twice/week	**1.91 (1.04–3.49)**	1.89 (0.99**–**3.61)	**1.93 (1.00–3.71)**
More than 3 times/week	**1.93 (1.52–2.45)**	**1.59 (1.20–2.10)**	**1.63 (1.23–2.16)**

*K6 (odds ratio to moderate psychological distress)*			
Less than once/week	Ref	Ref	Ref
Once or twice/week	1.00 (0.51**–**1.93)	1.06 (0.52**–**2.17)	1.08 (0.53**–**2.21)
More than 3 times/week	**0.77 (0.59–0.99)**	0.94 (0.69**–**1.28)	0.97 (0.71**–**1.34)

Bold numbers indicate *P* < 0.05.

a Adjusted for age and sex.

b Additional adjustment of model-1 for body mass index, educational level, subjective economic status, marital status, daily alcohol consumption, smoking status, subjective sleep quality, job type, hiring status, and average overtime hours.

c Additional adjustment of model-2 for physical activity and sedentary behavior.

For the K6 score, those who exercised three or more times a week showed a significant OR (OR = 0.77, 95% CI = 0.59–0.99, p = 0.044) in the model adjusted for age and sex; however, this significance was not observed after the adjustment for demographic variables in model 2 and model 3. Those who exercised twice per week showed no significant OR for a K6 score in any model. The analysis with not-imputed data showed similar results as imputed data (Supplementary Table 1). A slight difference in the significant results appears to be due to improved power in the imputed dataset.

In the stratified analysis by job type, there were positive associations between vigor of work engagement and workplace exercise among office clerks and administrative and professional workers similar to the results of the analysis in the overall participants (Table 4). Conversely, no significant results were observed among the service and sales workers.

**Table 4:** Comparison of work engagement and psychological distress between the groups; stratified by job type.

Frequency of workplace exercise	Model 1^a^	Model 2^b^	Model 3^c^
	OR 95% CI	OR 95% CI	OR 95% CI
*Office clerk (n = 520)*			
*Work engagement (odds ratio to high vigor)*			
Less than once/week	Ref	Ref	Ref
More than once/week	**1.59 (1.10–2.30)**	**1.54 (1.03–2.30)**	**1.64 (1.08–2.49)**
*K6 (odds ratio to moderate psychological distress)*			
Less than once/week	Ref	Ref	Ref
More than once/week	0.91 (0.62–1.33)	0.99 (0.65–1.51)	1.04 (0.67–1.60)

*Service or sales (n = 436)*			
*Work engagement (odds ratio to high vigor)*			
Less than once/week	Ref	Ref	Ref
More than once/week	1.29 (0.73–2.28)	1.63 (0.86–3.07)	1.63 (0.86–3.07)

*K6 (odds ratio to moderate psychological distress)*			
Less than once/week	Ref	Ref	Ref
More than once/week	0.86 (0.47–1.58)	0.78 (0.40–1.54)	0.81 (0.41–1.62)

*Administrative and professional (n = 334)*			
*Work engagement (odds ratio to high vigor)*			
Less than once/week	Ref	Ref	Ref
More than once/week	**2.61 (1.58–4.31)**	**2.03 (1.15–3.60)**	**2.07 (1.15–3.74)**

*K6 (odds ratio to moderate psychological distress)*			
Less than once/week	Ref	Ref	Ref
More than once/week	0.63 (0.34**–**1.17)	0.82 (0.39**–**1.73)	0.82 (0.39**–**1.74)

Bold numbers indicate *P* < 0.05.

a Adjusted for age and sex.

b Additional adjustment of model-1 for body mass index, educational level, subjective economic status, marital status, daily alcohol consumption, smoking status, subjective sleep quality, job type (except for analysis in the office clerk and service or sales), hiring status, and average overtime hours.

c Additional adjustment of model-2 for physical activity and sedentary behavior.

## 4 Discussion

This is the first study to examine how the frequency of workplace exercise was related to vigor of work engagement or psychological distress in a practical setting. The results indicate that a higher frequency of workplace exercise is related to higher vigor of work engagement independently from PA and SB. With psychological distress, workplace exercise did not show a significant relationship after adjustment for demographic variables. These findings suggest that workplace exercise with a frequency of at least once a week in practical settings improves employee vigor but not psychological distress.

Regarding the vigor for work in this study, the practice of workplace exercise showed a significantly higher OR in every model. Some interventional studies (Michishita et al., 2017, Strijk et al., 2013) that conducted workplace exercise programs reported a positive effect of the program on the vigor of work engagement. This study supports these results from the point of view of a non-interventional study that included a variety of participants. Other previous studies reported that workplace exercise improved work-related social capital compared to home exercise (Andersen et al., 2015), so that performing workplace exercise could enhance the resources of work engagement, such as social support at work (Schaufeli and Bakker, 2004). Additionally, social support is an important resource of work engagement not only at the individual level but also at the team level (Torrente et al., 2012), indicating that conducting workplace exercise with co-workers may enhance team-level work engagement. On the other hand, participants who performed workplace exercise three or more times a week showed slightly higher PA and less SB (10–30 min). Although increased PA and decreased SB seem to be induced by performing workplace exercise, the exercise itself could have a direct effect on improving employees’ work-related vigor because there was an independent association between these factors, regardless of the total amount of PA or SB. Moreover, performing workplace exercise once or twice a week showed a positive association with vigor of work engagement, thereby workplace exercise might have a specific effect on vigor in addition to increasing PA or decreasing SB. Although previous studies have reported that PA and SB are factors related to work engagement (Ishii et al., 2018, Munir et al., 2015, Nishi et al., 2017), providing workplace exercise may not necessarily enhance PA or decrease SB, given the specific effect of the exercise itself. Possible suggestions for exercise types at the workplace can be unsupervised programs such as “radio exercise (national calisthenics in Japan)” or stretching lasting for around 10 min, both of which are currently in use by many companies in Japan (Japan Sports Agency, 2017).

Job-type differences in work engagement in relation to PA or SB has been reported by Mullane et al. (2017); however, the complex association between these factors remain unclear. Non-service or sales (i.e., white-collar) workers displayed a positive association between workplace exercise and vigor of work engagement, whereas this relationship was not significant for service or sales. In contrast to service or sales workers, non-service workers do not interact with customers as frequently and tend to be sedentary and to stay at a specific location, such as a desk for extended periods (Smith et al., 2016). Extended periods of remaining seated is associated with reduced face-to-face interaction (Sugiyama et al., 2019). These differences in workstyle may modulate the effect of workplace exercise. Accordingly, the specific effects of a workplace exercise program might depend upon job type.

In terms of exercise frequency, previous intervention studies conducted exercise program either three to four times (Michishita et al., 2017) or once (Strijk et al., 2013) a week and confirmed the improvement of work engagement. In this study, participants who performed the exercise more than once a week showed a significantly higher OR of vigor of work engagement independently from PA and SB. Our results suggest that the frequency of workplace exercise for the improvement of vigor does not need to be three or more times a week. However, from a practical point of view, a workplace exercise frequency of three or more times a week is reasonable for improving work engagement because many participants performed the exercise three or more times (56.8%) over once or twice a week (4.2%). Although further study is needed, a frequent program might be effective to promote the habit of exercise without discontinuation or dropouts. Because organizational culture or support (e.g., management or leadership for health promotion, enhancing coworkers’ communication, setting an organization’s mission statement or policies) could be essential elements for successful workplace intervention (Taylor et al., 2018), enhancing these factors is important when implementing such programs.

In terms of psychological distress, the frequency of workplace exercise did not show a significant OR after adjusting for covariates. A systematic review of workplace PA interventions for mental health outcomes (Chu et al., 2014) reported no effect on mental health outcomes although the programs were held three or more times per week. Based on those results, the authors suggested that the effectiveness of interventions depended on the details of the exercise program, such as length, intensity, type of the exercise, and tailoring the program. Although the details of workplace exercise were not assessed in this study, some exercise conditions were not enough to improve psychological distress. Schaufeli et al. (2008) reported that work engagement is positively related to mental health. Therefore, improvement of work engagement in this study might lead to the improvement of psychological distress in the future. Future studies should investigate the details of workplace exercise (exercise frequency, intensity, time, and type) and conduct longitudinal studies on reciprocal relationships of work engagement and psychological distress to confirm these speculations.

Our findings provide support for current national movements in Japan. Recently, the Ministry of Economy, Trade, and Industry in Japan launched the new strategy “Health and Productivity Management,” an approach that considers investing in employees’ health as a corporate philosophy that should invigorate companies by improving employees’ vitality and productivity (Ministry of Economy Trade and Industry, 2018). Additionally, the Japan Sports Agency has started the “Sports Yell Company certification program” (Japan Sports Agency, 2017), which aims to promote companies that provide an opportunity for workers to have an active life via various practices (i.e., conducting workplace exercises, etc.). These strategies and certifications support health promotion at the workplace through company management and is anticipated to also benefit stock performance (Goetzel et al., 2016), healthcare cost, and enhanced productivity of workers. We hope that our findings will motivate workplaces to adopt workplace exercise to enhance work engagement.

This study has several limitations. First, because study data were obtained at the health checkup, details of work engagement (dedication, absorption) were not obtained due to limited space on the questionnaire. A previous systematic review (Knight et al., 2017) reported that the intervention effect of workplace exercise on work engagement assessed using sub-components of the scale was different from that assessed using the overall scale. Therefore, future studies are needed to clarify whether results from the sub-components show similar results from the overall scale. Second, because this was a cross-sectional study, we could not reveal causal relationships between workplace exercise and work engagement or psychological distress. Third, because the frequency of workplace exercise was evaluated using an unvalidated questionnaire, participants’ responses may vary from their actual practice. Finally, because the present participants of this study belonged to a single health insurance society in Tokyo and its surrounding areas, generalizing the results of this study should be done cautiously. We anticipate that numerous companies in various countries may introduce workplace exercise and hope that future research will be conducted to further investigate its effects, both positive and negative.

## 5 Conclusion

The practice of workplace exercise is positively related with the vigor of work engagement independently from PA and SB, and the frequency of workplace exercise might need to be at least once a week for the intervention program. On the other hand, only non-service or sales workers showed positive association, which indicates that differences in job type would modulate the association between vigor and workplace exercise. The workplace exercise, however, is not related to psychological distress in employees.

## Declaration of Competing Interest

The authors declare that they have no known competing financial interests or personal relationships that could have appeared to influence the work reported in this paper.
