# Supplementary material for: Relationship of workplace exercise with work engagement and psychological distress in employees: A cross-sectional study from the MYLS study
Source: Prev Med Rep. 2019 Dec 9;17:101030. doi: 10.1016/j.pmedr.2019.101030 (PMC6931185; doi:10.1016/j.pmedr.2019.101030)
Supplement: Supplementary data 1 [file mmc1.docx]

Supplementary Table 1. Comparison of work engagement and psychological distress between the groups by using not-imputed data

Bold numbers indicate *P* < 0.05. ^a^Adjusted for age and sex. ^B^Additional adjustment of model-1 for body mass index, educational level, subjective economic status, marital status, daily alcohol consumption, smoking status, subjective sleep quality, job type, hiring status, and average overtime hours. ^c^Additional adjustment of model-2 for physical activity and sedentary behavior.
